# Novel Mechanical Strain Characterization of Ventilated *ex vivo* Porcine and Murine Lung using Digital Image Correlation

**DOI:** 10.3389/fphys.2020.600492

**Published:** 2020-12-04

**Authors:** Crystal A. Mariano, Samaneh Sattari, Mohammad Maghsoudi-Ganjeh, Mehrzad Tartibi, David D. Lo, Mona Eskandari

**Affiliations:** ^1^ Department of Mechanical Engineering, University of California, Riverside, Riverside, CA, United States; ^2^ Delbeat LLC, San Francisco, CA, United States; ^3^ Division of Biomedical Sciences, School of Medicine, University of California, Riverside, Riverside, CA, United States; ^4^ BREATHE Center, School of Medicine, University of California, Riverside, Riverside, CA, United States; ^5^ Department of Bioengineering, Riverside, CA, United States

**Keywords:** biomechanics, digital image correlation, strain, deformation, pulmonary mechanics, lung, anisotropy, heterogeneity

## Abstract

Respiratory illnesses, such as bronchitis, emphysema, asthma, and COVID-19, substantially remodel lung tissue, deteriorate function, and culminate in a compromised breathing ability. Yet, the structural mechanics of the lung is significantly understudied. Classical pressure-volume air or saline inflation studies of the lung have attempted to characterize the organ’s elasticity and compliance, measuring deviatory responses in diseased states; however, these investigations are exclusively limited to the bulk composite or global response of the entire lung and disregard local expansion and stretch phenomena within the lung lobes, overlooking potentially valuable physiological insights, as particularly related to mechanical ventilation. Here, we present a method to collect the first non-contact, full-field deformation measures of *ex vivo* porcine and murine lungs and interface with a pressure-volume ventilation system to investigate lung behavior in real time. We share preliminary observations of heterogeneous and anisotropic strain distributions of the parenchymal surface, associative pressure-volume-strain loading dependencies during continuous loading, and consider the influence of inflation rate and maximum volume. This study serves as a crucial basis for future works to comprehensively characterize the regional response of the lung across various species, link local strains to global lung mechanics, examine the effect of breathing frequencies and volumes, investigate deformation gradients and evolutionary behaviors during breathing, and contrast healthy and pathological states. Measurements collected in this framework ultimately aim to inform predictive computational models and enable the effective development of ventilators and early diagnostic strategies.

## Introduction

Lung diseases are prevalent throughout the world causing 4.5 million deaths annually and imposing significant economic burdens and life quality strains on societies (Berman, 1991). Such pulmonary imposed circumstances are presently escalated in the recent respiratory-borne COVID-19 pandemic causing over 460,000 deaths with over 8 million confirmed cases worldwide as of July 2020 (Almukhtar et al., 2020; World Health Organization, 2020). Lung pathologies, such as asthma, emphysema, and chronic obstructive pulmonary disease (COPD), often alter the structural properties of lung tissue, where smoking or air pollutants trigger inflammatory cell recruitment that subsequently induces fibrosis (Li et al., 1999; Eskandari et al., 2013; Peng et al., 2018) and leads to irreversible damage as the tissue remodels (James and Wenzel, 2007; Eskandari et al., 2015, 2016). Induced lung dysfunction in asthma is associated with the loss of elasticity, airway narrowing, and obstruction in the lung (Gelb et al., 2002; Eskandari et al., 2015); hamsters with induced emphysema experience a lower elastic recoil pressure (Niewoehner and Kleinerman, 1973); and COPD lungs exhibit both decreased lung distension and reduced anisotropic tissue behavior (Pan et al., 2020). These studies demonstrate the pivotal role of mechanical properties in healthy and diseased lung function and highlight the importance of conducting fundamental pulmonary research.

The hierarchical nature of the lung from the organ to tissue to microstructure demands connective research across the scales (Mitzner, 2011; Suki and Bates, 2011). Early organ-scale studies have investigated the volume-pressure characteristic of the lung in saline (Ardila et al., 1974; Hoppin et al., 1975; Forrest, 1976) and air filled pulmonary specimens (Bayliss and Robertson, 1938; Mead et al., 1957). These works have been siloed from the material characterization of the airways (Eskandari et al., 2018) and parenchyma (Stamenovic and Yager, 1988; Suki et al., 2011; Birzle et al., 2019) at the tissue (Suki et al., 2005) or even microstructural scale (Eskandari et al., 2019). Furthermore, recent studies have recognized the important interplay between local tissue strains as it relates to mechanical overventilation in acute respiratory distress syndrome (Hurtado et al., 2020). To address these highlighted scientific gaps, we present the first, to the best of our knowledge, full-field deformation characterization of the *ex vivo* whole lung.

Here, we introduce a method of associating pulmonary scales and linking measurements of local tissue strain and deformation behavior to the bulk, classical organ volume-pressure global response. Specifically, we have interfaced a Digital Image Correlation (DIC) set up with our recently developed novel volume-pressure inflation/deflation apparatus (Sattari et al., 2020). This enables investigations of deformations and strain topologies of small (murine) and large (porcine) animal model lungs under highly controlled inflation-deflation cycles, where the continuous evolutionary and gradient response and the role of maximum volume and flow rate can also be examined for the first time.

While DIC has proven an effective technique for studying biological tissues such as ligaments (Mallett and Arruda, 2017), tendons (Luyckx et al., 2014), bones (Sztefek et al., 2010), arteries (Zhang et al., 2002), and the brain (Libertiaux et al., 2011), the rapid deformations in breathing and the complexity of handling lung tissue have restricted analogous advancements in pulmonary biomechanics; such measurements have not been reported until now. Our study enables the first analysis of strain values across the surface of a lung specimen, facilitates visualization of topological gradients to consider localized mechanical behavior, and yields novel insights regarding how the lung continuously elongates and expands during virtual breathing in real time.

This study serves as a proof-of-concept for future works to comprehensively characterize the regional response of the lung across various species, and contrast healthy and pathological states using DIC. We present the experimental methods used to properly measure expansive volume change of the lung using DIC for both murine and porcine animal models. The preliminary results of strain distribution patterns and associated volume-pressure behavior are discussed in light of the fundamental implications on lung spatial heterogeneity, anisotropy, and elastic properties as a function of maximum loading volumes and rates. The seminal results and new applications provided in this work offer a unique approach to study lung deformation, continuously connect organ response to localized behavior, and lay the foundation for significantly advancing pulmonary biomechanics research.

## Materials and Methods

### Sample Preparation

**Figure 1:**
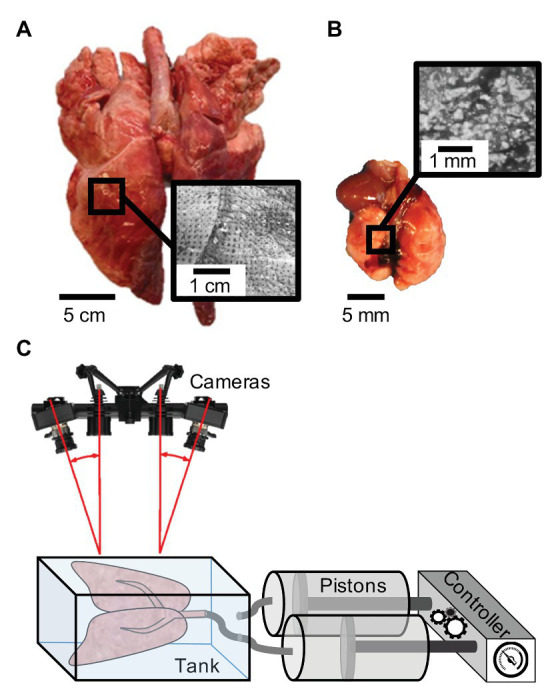
Specimen speckle pattern and experimental set-up: **(A)** Porcine sample (left) and **(B)** murine sample (right) with illustrated respective speckling patterns for digital image correlation (DIC). **(C)** Schematic of the uniquely constructed volume-pressure inflation system interfaced with the DIC Trilion ARAMIS cameras through use of a transparent tank with polarizers to analyze topological strain (Trilion Quality Systems GOM ARAMIS, 2016; Sattari et al., 2020).

Pulmonary specimens of two distinct size scales, a pig and mouse, were tested. For the porcine sample, a lung from a 50 kg female domestic York farm minipig (Figure 1A) was transported in a cooler and kept refrigerated until tested. Tests were completed within 48 hrs after sacrifice; the animal was part of a cardiovascular study with no pulmonary interference (Institutional Animal Care and Use Committee approval AUP#174424-02). A 10-week old C57BL/6J murine lung (Figure 1B) was collected from the University of California, Riverside’s School of Medicine (Institutional Animal Care and Use Committee approval, AUP#20180009). The mouse was tested immediately after sacrifice. Both porcine and murine samples were kept moistened prior to testing in 1X phosphate-buffered saline (PBS) solution.

### Specimen Inflation

A laboratory air-line pressure outlet of 40 psi was used to inflate the pig lung specimen utilizing a plastic tube inserted through the trachea. The lung was preconditioned for a total of eight times, including its initial inflation to open its airways, prior to analyzing the final response. Preconditioning is a necessary step for stabilizing biological materials and to enable consistent and reproducible outcomes (Cheng et al., 2009; Terry et al., 2014). The porcine sample was actively inflated for 70 s and deflated by detaching the tube to passively excavate air for the remaining 70 s. The large topology of the pig lung was primarily utilized for considering heterogeneous and anisotropic strain behaviors and was not linked to pressure or volume values.

The mouse lung was used to associate measures of organ pressure, maximum volume, and rate dependent behaviors. Inflation and deflation tests were conducted using a custom-designed pressure-volume system (Sattari et al., 2020). This programmable apparatus (Figure 1C) consists of a controller and pistons interacting with the DIC setup through a transparent tank to associate measured strains and lung pressures to the applied volume and rates. Using the volume-pressure system, we designated 10 cycles of preconditioning for each inflation volume and each flow rate with a tare load of 0.05 psi. After preconditioning, a single inflation-deflation cycle (actively pushing air both in and out) was analyzed, followed by a hold of 80 s. The viscoelastic hold was implemented to assess if strain measurements were quasi-static or a function of air-flow redistribution in the tissue. Rate dependency was considered by inflating to 0.5 ml at rates of 0.1 and 0.02 ml/s. Potential volume thresholds and physiological limits were also explored by examining different maximum volumes of 0.3, 0.5, 0.7, and 0.9 ml at a fixed rate of 0.1 ml/s.

### Digital Image Correlation

The basic working principles of the DIC technique involved creating a random pattern of features on the surface of a sample that undergo arbitrary deformation (Palanca et al., 2016). The system used that pattern to track the points in motion by taking still images with facets to observe and match to the deformed state while computing the points in three dimensions (3D). The points on the surface were used to measure the displacement and compute strain (Trilion Quality Systems GOM ARAMIS, 2016). DIC’s advantages included the study of whole lung deformation continuously and in real time without the need for extensive sample preparation steps, which often alter the intact tissue properties, or image processing techniques required for digital volume correlation material (Arora et al., 2017).

We used the Trilion ARAMIS Adjustable 12M system, with blue-light filters and polarizers (Trilion Quality Systems, King of Prussia, Pennsylvania) to track the distension of the pig and mouse specimens over the inflation and deflation periods. The two-camera stereo system (Figure 1C) with 4,096 × 3,000 pixels resolution were calibrated to the measuring volume of either the pig or mouse lung to allow for precise tracking (Trilion Quality Systems GOM ARAMIS, 2018a). A facet size of 25 and 30 pixels and point distance of 10 and 15 pixels were used for the larger pig and smaller mouse lung tests, respectively; for example, on the pig lobe, this resulted in more than 8,700 data points. Preliminary facet size sensitivity was conducted, assessing a reproducible number of pattern points within each facet, controlling for measurement point density throughout the duration of the test and avoiding data loss due to motion (Trilion Quality Systems GOM ARAMIS, 2018b). As multiple measurement points were calculated, a boundary outlining the lung was created in order to generate a full surface component allowing the software to track deformation and calculate strain.

### Speckle Patterning

In order to achieve the stochastic speckling pattern central to DIC tracking, we found that the specimen must be speckled in the expanded inflated position to ensure visibility of all surfaces for the duration of testing. For example, the porcine specimen commonly doubled in volume from the deflated state where there were crevices and non-visible regions. The moisture on the lung surface created a glare, leading to blind spots during tracking; therefore, porcine and murine specimens were both gently blotted to matte the surface and polarizers were used on the cameras to alleviate this effect. Additional complications specific to lung tissue is the drastic change in color from dark pink to light blush, problematic to the visual contrast needed in DIC. To remedy this issue, quick-drying white enamel paint (rust-oleum) was lightly sprayed onto the pig sample using the aerosol spray can, as in other DIC methods (Zhang et al., 2002; Sztefek et al., 2010). Next, a generic silicone exfoliator pad was used to apply the Gentian Violet dye on the porcine specimen. Among many evaluated applicators, the exfoliator pad was optimal in creating a sufficient stamped dot size for the pig lung (Figure 1A).

The speckling method for the pig lung was too large for the mice; therefore, we utilized an alternative method similar to Mallett and Arruda (2017). A thin layer of water-resistant hybrid paint (ProAiir Makeup, Knoxville, Tennessee) was sprayed at a 10 cm distance for 90 s using the Infinity CR 2 airbrush (Harder & Steenbeck, Brooksville, Florida) with a 0.2 mm nozzle (Shi et al., 2019). The same paint was filled into a generic spray bottle used at a 20 cm distance for approximately 50 sprays to create larger, distinct dots for DIC (Figure 1B). Unlike the porcine specimen, our preliminary assessments found that the white water-resistant paint created sufficient contrast in the mice lungs.

### Data Collection and Analysis

The DIC measurements on both pig and mouse samples were conducted using the ARAMIS camera system and analyzed using the GOM software (Trilion Quality Systems GOM ARAMIS, 2018b). The data collection frequency ranged from 2 to 10 Hz, adjusting to the time duration of various species’ and rates’ inflation-deflation test cycles. Locations (labeled letters) on the surface of the lungs were selected for analysis, scattered in a nearly equidistant formation to represent various regions and loads, and technical strain measurements were computed from the undeformed, uninflated initial state. The major and minor strain directions and magnitudes were calculated to evaluate maximum stretches and heterogeneous stretch distributions; additionally, the ratio of minor to major strain was used to assess the anisotropy of the lung, where a ratio of 1.0 indicated a pure isotropic response. Selected strain points from the lung surface were associated to the volume and pressure measurements from the inflation apparatus. Strain vs. volume and strain vs. pressure curves were plotted using the same range to analyze the various regions of the lung lobes; these graphs enabled compliance comparisons associated with tissue elasticity (Sera et al., 2004), where a greater slope and steeper rise in strain-volume or strain-pressure plots indicated higher tissue compliance. Inversely, decreased slopes meant greater tissue stiffness (Okamoto et al., 2014) as there was more resistance to inflation.

## Results

### Porcine Study

**Figure 2:**
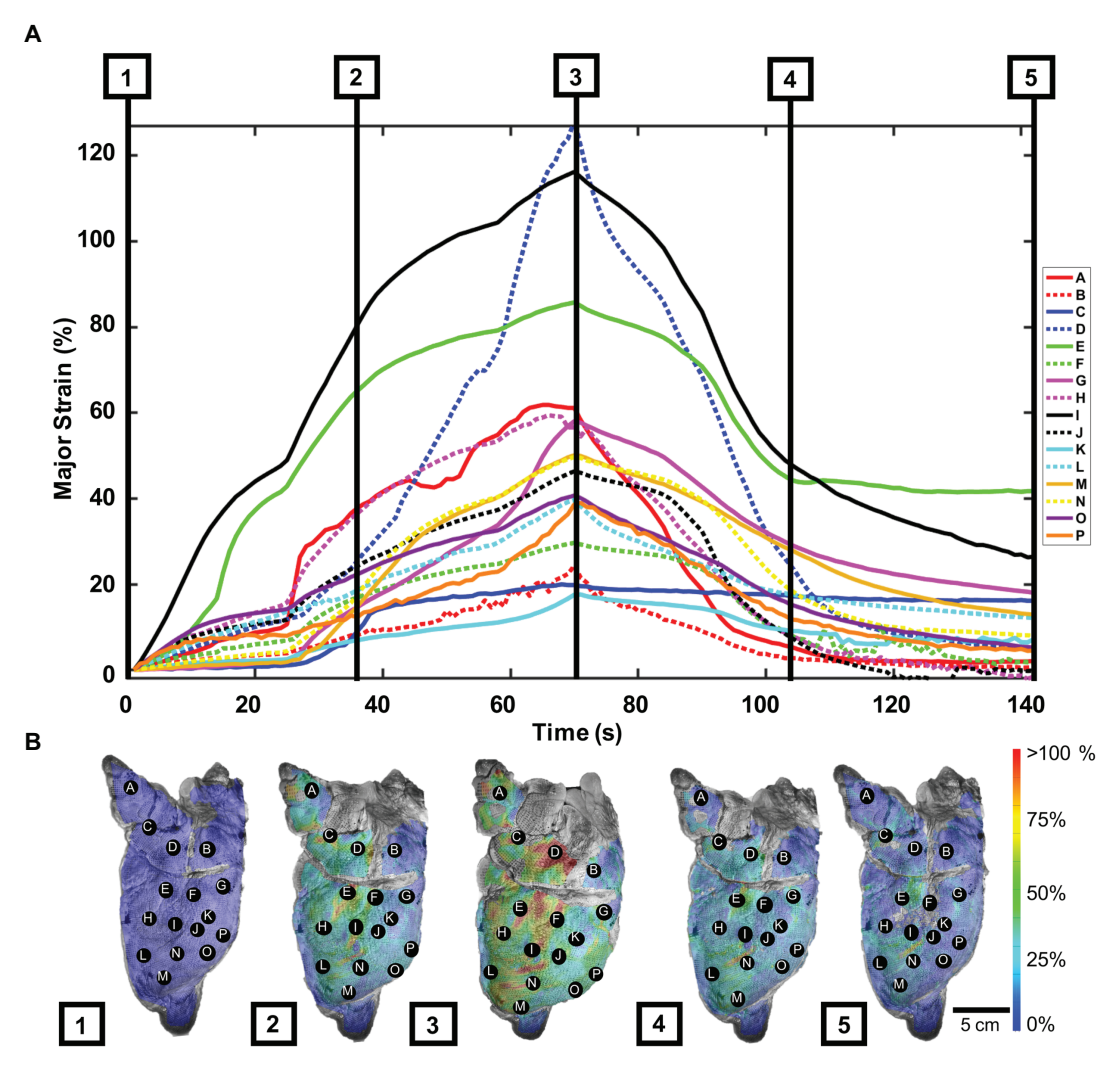
Porcine major strain over time: **(A)** Strain values as labeled points across the left pig lung lobe during five phases of the inflation-deflation cycle: initial state **(1)**, mid-inflation **(2)**, maximum inflation **(3)**, mid-deflation **(4)**, and end state **(5)**. **(B)** Strain color map images corresponding to the five cycle phases relayed deformation heterogeneity. A–D were located in the cranial lobe, while points E–P were scattered in the caudal lobe (Madan and Fan, 2018).

Strain values by labeled lettered points and contour maps of the porcine lung were plotted over the inflation-deflation cycle (Figure 2). Generally, all points A–P increased in strain over time during inflation, yet the rate of strain increase and peak strain value range greatly differed depending on the spatial location (Figure 2A). For instance, point D was less responsive to inflation than point I, but then rapidly stretched until its peak value surpassed that of point I. At peak inflation, strains ranged in value from 17.2 to 128.1%. The passive elastic recoil of the lung, when the air was no longer pumped into the tissue, demonstrated faster deflation rates (steeper negative slopes) than during active inflation, specifically in the first 30 s.

Figure 2B showed porcine topological strain contours captured at five inflation-deflation cycle time points: initial state, mid-inflation, maximum inflation, mid-deflation, and end state. The lung drastically deformed at inflation phases 1–3, particularly in the central region, where the highest strain was seen (points D, E, and I). The lung was noted to not fully reset to its original state after 70 s, when the air was left to passively escape from the trachea; instead, the strain lingered in areas of high strain.

**Figure 3:**
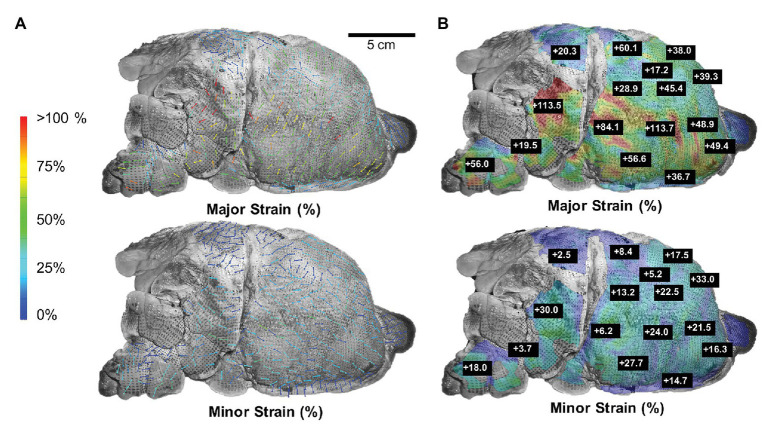
Major strain vs. minor strain: **(A)** Directional analysis of major (top) and minor (bottom) strain with generally larger expansion trends pointed in the lateral-medial and ventral-dorsal direction compared to cranial-caudal. **(B)** Peak inflation strain surface color map comparison of major and minor strain exhibited region-varying anisotropic behavior.

At peak inflation, Figure 3 illustrated major and minor strain directions along with corresponding major and minor strain values of the lettered location labels. Major strains dominantly aligned with lateral-medial and ventral-dorsal directions, while minor strains oriented with cranial-caudal directionality (Figure 3A). The minor strain values, calculated at matching points informing major strain, ranged 2.5–33.0% (Figure 3B). The anisotropic ratio varied from 0.07 to 0.84, with the greatest anisotropy at point E and near isotropy at point O.

### Murine Study

**Figure 4:**
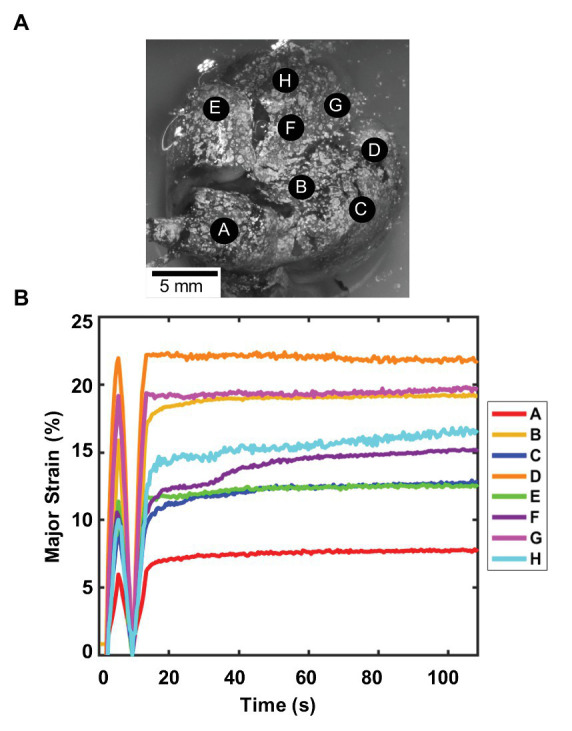
Murine major strain vs. time: **(A)** Spatial locations considered for strain analysis. **(B)** Strain over time of one inflation-deflation cycle, followed by inflation-hold for viscoelastic considerations found small changes in strain values demonstrative of quasi-static behavior and minimum impact of flow on the system. Point A is located on the right superior lobe, E is on the middle lobe, and points F, H, and G are on the right inferior lobe. For the left lung, point B is on the left superior lobe, and C and D are on the left inferior lobe (Cardona et al., 2000; Ellis et al., 2003). Regional strain heterogeneity is noted as strains differed in range by 15%.

Unlike the porcine natural deflation response, the murine lung was inflated and deflated to the same volume at a constant known rate using the volume-pressure piston apparatus. While the smaller lung specimen challenged surface analysis trends, Figure 4A showed the location of several representative lobe points A–H used to report the strain-time response of the mouse. The last inflation-deflation test cycle prior to the 80 s hold was shown in Figure 4B. As 0.5 ml air was pushed into the lung at 0.1 ml/s, all points A–H increased in strain values, with peak values 6–21%. During the subsequent inflation and hold cycle, points measured strains of 7–22% and held nearly steady for all points over time, demonstrative of the quasi-static nature of the measurements, enabling association of the pressure measures to internal stresses in the tissue according to Pascal’s law; this approach was in agreement with previous studies, which found that at slow enough inflation, lung mechanics is not impacted by flow (Bayliss and Robertson, 1938; Mount, 1955).

**Figure 5:**
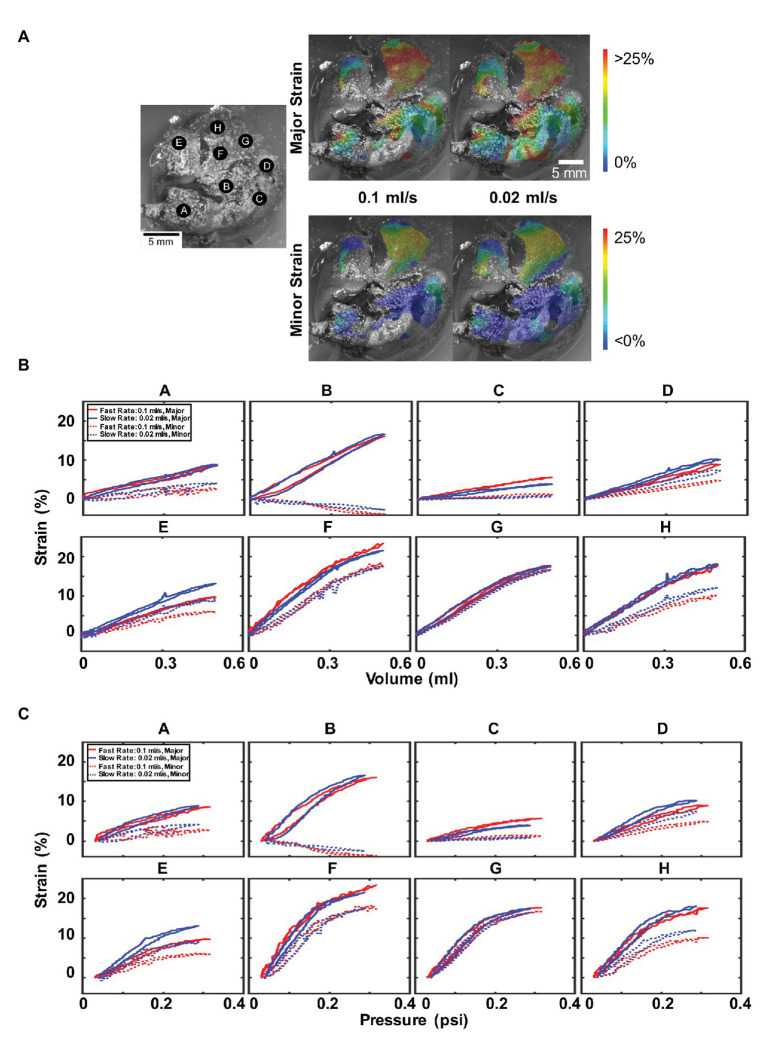
Effect of inflation rate: **(A)** Topological strain color map for major and minor strain at fast (0.1 ml/s) and slow (0.02 ml/s) inflation rates. **(B)** Major and minor strain locations plotted for 0.5 ml inflation volume and similarly, **(C)** major and minor strains vs. pressure. Faster inflation rates result in higher peak pressures but do not necessarily result in higher strains. Anisotropy and heterogeneity were observed in addition to a non-linear and changing relationship between strains, volumes, and pressures.

Figure 5 illustrated the major and minor strain contours (Figure 5A), and specific strain-volume (Figure 5B) and strain-pressure (Figure 5C) relationships by lettered locations for 0.5 ml inflation at fast (0.1 ml/s) and slow rates (0.02 ml/s). Topological strain color maps found the right inferior lobe (F, H, and G) collected the highest strains for both major and minor directions and for both rates. The inflation and deflation closed-loop cycle for strains related to both volume or pressure, differed in behavior for nearly every location A–H: slower rates were seen to produce greater major strain values at locations D and E, while faster rates produced greater major strains for points C and F, and negligible rate dependencies were observed elsewhere.

Interestingly, uniform across all locations was the observed greater peak pressure values reached for the faster inflation rate. Additionally, location B seemingly exhibited uniaxial stretch, with elongation in the major strain coupled to compression in the minor strain; it was also noticed to have the most hysteresis. On the other hand, point G had major and minor strains that coincide and indicated isotropic strain.

Furthermore, most strain-volume behaviors were linear and constant (Figure 5B); however, notable nonlinearity was observed for points F, G, and H, corresponding to the highest strains on the right inferior lung lobe. When the volume increased beyond ~0.3 ml, both major and minor strains did not increase at the same slope, but a decreased strain rate was noticed. This nonlinearity was more prominent in the corresponding strain-pressure graphs (Figure 5C), where points B, D, and E–H were bilinear, with drastically changed slope at ~0.2 psi pressure.

The murine anisotropy ratios at each location were calculated at their peak inflation point for the fast and slow inflation rates (Table 1). Regions drastically varied with their anisotropic absolute value ratios between 0.13 and 0.94 for both rates. For both fast and slow rates, the same lettered locations (C and G) depicted the highest and lowest anisotropic ratio values.

**Table 1:** The minor to major anisotropic strain ratio for parenchymal mouse lung regions at 0.5 ml for fast and slow inflation rates.

	A	B	C	D	E	F	G	H
0.1 ml/s	0.31	−0.24	0.20	0.54	0.59	0.77	0.94	0.58
0.02 ml/s	0.32	−0.21	0.13	0.63	0.67	0.66	0.87	0.56

Most locations had strong anisotropy and strained 2–3 times more in the major than in the minor direction. The effect of loading rate does not appear to uniformly impact the anisotropic response, with decreased ratios for points A, D, and E for 0.1 ml/s, but with increased ratios other locations.

**Figure 6:**
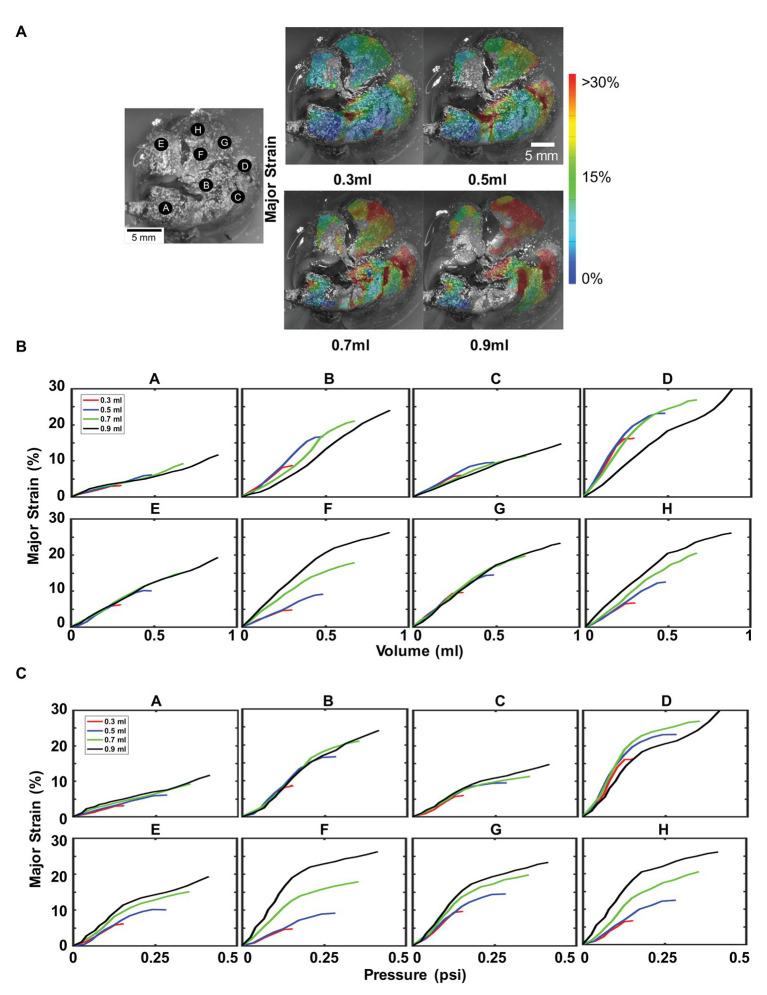
Effect of maximum inflation volumes: Inflation loading for multiple maximum volumes at 0.1 ml/s was shown, labeled A–H as in Figure 3. **(A)** Topological surface strains showed an increase in strain caused by an increase in maximum inflation volume. **(B)** Major strain versus volume, demonstrated region dependent behavior. **(C)** Effect of increasing maximum inflation volume on lung pressure led to disparate tissue expansion trajectories, mainly increased compliance (increased slope).

Figure 6 illustrated the topological strains, strain-volume, and strain-pressure relationships for four maximum inflation volumes of 0.3, 0.5, 0.7, and 0.9 ml at a constant rate of 0.1 ml/s (deflation cycle not shown for visual clarity). Strains beyond 30% appeared in both the right and left inferior lobes, mainly for 0.7 and 0.9 ml inflation volumes (Figure 6A). Locations A, C, E, and G demonstrated increased strains for increased volumes, maintaining the same unidirectional relationship and slope regardless of the amount of inflation volume (Figure 6B). Points B and D found that for increasing volume (0.7 and 0.9 ml), the strain did not increase as rapidly as it had for the smaller volumes (0.3 and 0.5 ml), instead decreasing in slope. However, locations F and H found the strain to increase more rapidly for larger inflation volumes, increasing in compliance. The effect of maximum volume on the strain-pressure behavior was more pronounced (Figure 6C): for most locations, the slope of the strain-pressure response increased for larger inflation volumes (B and D were the only two exceptions).

For larger volumes (0.7 and 0.9 ml), there is an inflation threshold, beyond which the change of pulmonary surface expansion decreases. This notable nonlinearity and transition from a higher to lower slope denoted modified material compliance, specifically, increased material stiffness. For instance for 0.9 ml, nearly all lettered locations experienced a decrease in slope at ~0.5 ml volume (Figure 6B). Corresponding pressure graphs for locations B–H (Figure 6C) observed a similar transition at ~0.12 psi. Lower maximum volumes (0.3 and 0.5 ml) did not exhibit this degree of nonlinearity and while this phenomenon was also seen in the flow rate tests (Figure 5), it remained unchanged for alternate flow rates.

## Discussion

### Surface Heterogeneity

The lung surface strain maps obtained for both porcine (Figures 2, 3) and murine (Figures 5, 6) lungs demonstrate marked heterogeneous expansion across regions of the lung. Pulmonary regional heterogeneity can be due to imbalanced regional ventilation (Milic-Emili et al., 1966; Grasso et al., 2008; Mitzner, 2011), the parenchymal behavior (Denny and Schroter, 2006), or the airway geometry and material (Mead et al., 1957; Arora et al., 2017; Eskandari et al., 2019). The centrally located regions of highest stress in the pig lung appear to trend with the trajectory of the main bronchi (Figure 2), suggesting ventilation in the airways to be a main contributor to heterogeneity; post-experiment transverse plane dicing of the porcine lung reinforced this notion. Such verification is not possible on the murine lung given the physical size limitations; however, the lower lobe appeared to conduct the largest strains in both varying volume and rate tests (Figures 5, 6).

The size and orientation of airways vary throughout the tissue (Weibel, 1960; Sinclair et al., 2007), promoting observed lung strain heterogeneity during ventilation, further enhanced by varying material properties, as seen with the increasing stiffness of distal airways (Eskandari et al., 2018). Our first observations in *ex vivo* murine and porcine lung find the lower and central regions exhibit greater strain unlike in humans, where the upper regions have greater expansion. While this may be due to the effect of gravity on the human upright position as opposed to prone stature (Milic-Emili et al., 1966), it may additionally be due to the difference in bronchial network morphology (monopodial vs. dichotomous).

The higher inflation rate color map for the mouse lung (Figure 5A) was anticipated to exhibit higher, more concentrated strains compared to the slower inflation rate due to the latter having more time to equilibrate and disperse air throughout the lobes. From this limited study, conclusions about the strain heterogeneity due to rate were elusive; however, these characteristic regional differences support the idea of the lung operating asynchronously (Otis et al., 1956).

Evaluating spatial heterogeneity of the lung may have important implications for clinical applications, such as optimal surgical planning and efficient ventilation. Previous studies have highlighted that decisions regarding the extent of resection in patients with early-stage lung cancer would benefit from incorporating lung function needs as measured by forced expiratory volume in 1 s (FEV1), (Gu et al., 2018); similarly, understanding the local spatial strains can inform the region-specific expansion role and be weighed against surgical needs. Additionally, documenting healthy strain local tissue deformation behavior can help prevent ventilator induced lung injury by assessing overstretch thresholds based on expected regional trends; the performance of recently proposed multi-frequency rotation ventilation machines hinges on this knowledge of local lung deformation in order to efficiently accommodate all regions without causing major damage to the lung (Hager et al., 2007; Herrmann et al., 2016).

### Lung Anisotropy

Strain anisotropy, as defined by the ratio of minor to major strain with unity defining isotropy, differed across the tissue landscape with some locations more isotropic than others in both the porcine and murine lung. Additionally, the degree of anisotropy evolved within each location: changes to the flow rate documented varying degrees of anisotropy even though the maximum volume inflation was identical (Table 1). The divergent major and minor strain loading trajectories convey increasing anisotropy for increasing inflation volume (Figure 5). This whole organ surface strain anisotropy can emanate from a combined effect of non-uniform loading due to the complex underlying bronchi network (Weibel, 1960; Tawhai et al., 2004; Oakes et al., 2012), the highly curved shape of the lung lobe geometry (Wagner et al., 1967; Powell et al., 2016), and parenchyma and airway material anisotropy (Mitzner, 2011; Eskandari et al., 2018).

Previous studies have demonstrated that airway constituents, such as intertwined collagen and elastin, give rise to direction dependent mechanical properties of the airways being stiffer in the longitudinal direction compared to the circumferential direction (Bayliss and Robertson, 1938; Sera et al., 2004; Eskandari et al., 2019). Given the monopodial nature of the porcine and murine lungs, where a single main branch extends through the central right and left lungs (Monteiro and Smith, 2014), the more compliant circumferential airway material may translate to the observed dominant lateral-medial and ventral-dorsal oriented major strain (Figure 3A). The current work in our lab is focused on documenting the surface strains for human lungs, which have dichotomous branching and may potentially yield more isotropic surface strains.

Knowledge of lung anisotropy is critical for developing accurate predictive lung computational models, which rely on realistic material properties and currently lack anisotropic considerations (Eskandari and Kuhl, 2015). In addition, anisotropy may also serve as a biomarker in the early detection of some lung diseases where the degree of anisotropy is adversely altered; for instance, COPD lungs exhibit lower anisotropic behavior correlating with the severity of COPD (Pan et al., 2020). Such insights can be exploited to assess healthy lung function and deviations from normal physiology.

### Changes in Tissue Compliance

Examination of the mouse lung by interfacing DIC with a ventilation apparatus enables association of recorded strains to inflation volumes and measured pressures. We observe a non-linear relationship and varying degrees of decreased lung compliance (Figures 5, 6). This decreased slope-shift in strain is likely due to the lung reaching its expansion limit (Jonson and Svantesson, 1999) and is indicative of a potential physiological threshold beyond which strain hardening is observed (Sinclair et al., 2007; Eskandari et al., 2018). Several regions of the mouse lung under fast and slow inflation rates exhibit this behavior (Figures 5A,B), indicating increasing resistance to expansion and stiffer response. Larger inflation volumes (0.7 and 0.9 ml) demonstrate this physiological threshold for most regions on the murine lung, yet the smaller inflation volumes (0.3 ml in particular) barely exhibit this altered compliance threshold (Figures 6A,B). The change in flow rate (assessed at 0.5 ml) did not necessarily impact the lung compliance alteration, but lower maximum volumes did not reach this physiological inflation limit to exhibit non-linearity, and therefore maintained compliance (and slope) throughout the test.

Faster inflation rates measured higher peak pressures for all lettered locations, indicative of pulmonary viscoelasticity (Sattari and Eskandari, 2020). Biological tissues are known for exhibiting similar rate-dependent behaviors: in brain tissue, it has been observed that faster rates would result in strain hardening and a stiffer response (Rashid et al., 2014). While we see increased peak pressures for faster inflation, the stiffening of the tissue with faster rate (Zhang et al., 2008), as anticipated by the decreased slope of the strain-volume or strain-pressure curve, varies depending on the location. This suggests that while the lung is viscoelastic and rate loading dependent, regions of the tissue may either increase or decrease in stiffness.

For all mouse lung locations A–H, the response to smaller air volumes 0.3 and 0.5 ml follow the same, near-linear trajectory (Figures 6A,B) and slope. However, 0.7 and 0.9 ml inflation tests exhibit predominantly increased strain-volume or strain-pressure slopes, indicating decreased resistance to expansion. Given that the estimated total lung capacity of a mouse is 1 ml (Irvin and Bates, 2003), it is possible that lower volumes did not have as many airway passages open and available to expand (Engel et al., 1975). Loading history dependency is also likely as air may stay trapped after subsequently loading from 0.3 to 0.9 ml (despite preconditioning for each new volume level). In locations where the earlier 0.5 ml inflation level exhibits the non-linear physiological threshold, the subsequent 0.7 and 0.9 ml tests conform to increased slope trajectories (Figure 6C, locations E, F, G, and H). Similar to the material altering concepts of yielding or plasticity in innate metals, once the lung reaches its expansion limit, more airways open and the subsequent tests experience easier to achieve and greater stretch levels for the same pressures (Naureckas et al., 1994). The experimental pulmonary strain measurement method presented here can have direct implications on studying pulmonary complications caused by harmful overstretching, such as ventilated induced lung injury; assessing the interplay between changing the flow rate or maximum inflation volumes may provide new strategies to hamper strains in the lung and improve ventilation strategies.

### Limitations

A general consideration in the measurement of lung inflation conducted in this study is the use of positive pressure inflation and explanted lung lobes. This is distinct from physiological lung distension under negative pleural pressures in the thoracic cavity. The lung is dependent on the combined expansion effects of the rib cage with contraction of the diaphragm to lower the floor of the thoracic cavity. Negative pressure inflation in pathologic settings can cause collapse of the airways and restrict airflow. However, the question is whether overall lobe tissue dynamics can be altered by the closed compartment of the pleural cavity under negative pressure. One indication that the three-dimensional geometry of the pleural cavity can significantly affect inflation is that pleural negative pressures during respiration are significantly reduced in patients after surgical lobe resection (Bok et al., 2018). Thus, changes in the way lung tissue fills the pleural cavity appear to change the negative pressures during breathing, and may significantly alter local tissue dynamics during inflation. This may possibly affect regional aeration of lung tissue.

A measure of lung aeration is an assessment of atelectatic (collapsed) lung tissue, and a comparison of positive vs. negative pressure inflation of perfused lungs found consistently higher percentage of atelectatic lung from positive vs. negative pressure ventilation (Grasso et al., 2008). It is possible that this effect is due to pressure gradients from the rib cage across the tissue as they interact with regional tissue elasticity under positive vs. negative pressure inflation. However, these considerations may be mainly of relevance only to regional aeration of lung tissue; other studies have confirmed that overall mechanical parameters are not significantly altered by inflating *ex vivo* lung tissue under negative vs. positive pressures (Peták et al., 2009). Thus, while differences in the experimental assessment of lung tissue dynamics may reveal localized effect on tissue inflation, the overall mechanics of the examined tissue remain intrinsic to the tissue itself. Nonetheless, the *ex vivo* approach conducted here enables explorations of highly-controlled and wide-range breathing rates and volumes, which would be too invasive for *in vivo* studies.

Conclusive inference regarding lobe strains is premature, given the proof-of-concept scope of this manuscript. However, insights gained in this study will be used to conjecture the use of surface strains to represent the projected strain in the bulk of the tissue. Measuring only topological strains *via* our DIC measurements means conclusions regarding the internal 3D strain distribution within the lung cannot be assessed. We are able to gain access to internal cues of the lung given our volume-pressure ventilation system or tensile tests on sections of the dissected tissue (Eskandari et al., 2018; Sattari et al., 2020). The measured surface strains will enable new comparative studies on the deformation characteristics of healthy and diseased lungs in the future, and conclusions regarding the comprehensive behavior of regional ventilation, airway network, and parenchymal matrix can be made from a pseudo-material response from lung surface behavior (Mitzner, 2011).

The objective of this study was to establish DIC as a tool for measuring pulmonary behavior on two alternate specimen scales. Results for limited samples are presented here and not intended as a statistically rigorous conclusion of either porcine or murine lung behavior. Our regional observations are mainly intended to illustrate approach validity and functionality. Future experiments will be conducted on statistically inductive numbers of specimens to incorporate inter-animal variability and enable the conclusion of the biomechanical properties exclusive to each species.

## Conclusion

In this manuscript, we explore the first capabilities of DIC on *ex vivo* murine and porcine specimens to investigate local strains, laying the foundation for future applications of lung characterization. Notable anisotropic and heterogeneous strain trends were observed on the surface of the lungs. Furthermore, the unique combination of 3D DIC with a custom-designed volume-pressure apparatus granted access to physiological pulmonary loading with versatile control over parameters such as flow rate and maximum volume inflation. To the best of our knowledge, this study is the first to report such whole-lung measurements and additionally associate continuous strain measures with classical volume-pressure tests with real time dependencies. While DIC has been used in the past to study other soft tissues, it has not been employed to investigate the lung at the organ level until now.

## Data Availability Statement

The raw data supporting the conclusions of this article will be made available by the authors, without undue reservation.

## Ethics Statement

The animal study was reviewed and approved by Institutional Animal Care and Use Committee approval AUP#174424-02 and AUP#20180009.

## Author Contributions

ME conceptualized and supervised research. CM and SS designed and performed experiments. DL and MT provided resources. CM, ME, MM-G, and MT interpreted results. CM, ME, and MM-G analyzed data and drafted the Figures. CM, DL, ME, and MM-G wrote the manuscript. All authors contributed to the article and approved the submitted version.

### Conflict of Interest

The authors declare that the research was conducted in the absence of any commercial or financial relationships that could be construed as a potential conflict of interest.
